# Belantamab mafodotin plus venetoclax in t(11;14)-positive relapsed or refractory multiple myeloma – the BELI(E)VE trial

**DOI:** 10.1186/s12885-026-16905-3

**Published:** 2026-09-16

**Authors:** Christoph Schaefers, Cyrus Khandanpour, Mathias Hänel, Miriam Kull, Winfried Alsdorf, Abdulaziz Kamili, Ricardo Kosch, William Krüger, Stephan Bohl, Elias Karl Mai, Christof Scheid, Hermann Einsele, Monika Brüggemann, Anna Suling, Antonia Zapf, Carsten Bokemeyer, Katja Weisel, Lisa Leypoldt

**Affiliations:** 1 Department of Hematology, Oncology and Bone Marrow Transplantation with Section Pneumology, University Medical Center Hamburg-Eppendorf, Martinistraße 52, Hamburg, 20246 Germany; 2 Department of Hematology and Oncology, University Hospital of Schleswig-Holstein Campus Lübeck, University Cancer Center Schleswig- Holstein and University of Lübeck, Lübeck, Germany; 3 Department of Hematology, Oncology and Bone Marrow Transplantation, Klinikum Chemnitz, Chemnitz, Germany; 4 Department III of Internal Medicine University Hospital of Ulm, Ulm, Germany; 5 Department of Internal Medicine C, University Medicine Greifswald, Greifswald, Germany; 6 Department of Internal Medicine, Charité—University Medicine Berlin, Berlin, Germany; 7 Heidelberg Myeloma Center, Internal Medicine V, Hematology, Oncology and Rheumatology, Heidelberg University Hospital and Medical Faculty Heidelberg, Heidelberg, Germany; 8 Department I of Internal Medicine, University Hospital of Cologne, Cologne, Germany; 9 Department of Internal Medicine II, University Hospital Würzburg, Würzburg, Germany; 10 Department of Hematology, University of Schleswig-Holstein, Campus Kiel, Kiel, Germany; 11 Institute of Medical Biometry and Epidemiology, University Medical Center Hamburg-Eppendorf, Hamburg, Germany

**Keywords:** Multiple myeloma, t(11;14), Belantamab mafodotin, Venetoclax, Trial protocol

## Abstract

**Background:**

Multiple myeloma (MM) is a widely incurable B-cell malignancy that predominantly affects older adults, representing 10% of hematologic cancers. Despite advancements in therapy that have significantly extended median overall survival (OS), MM remains a clinical challenge with poor prognosis for patients refractory to multiple drug classes. There is a critical need for innovative and personalized treatments, including for patients with translocation (11;14), a subgroup characterized by specific therapeutic vulnerabilities. Combined targeting of B-cell maturation antigen (BCMA) and BCL-2 presents a promising personalized treatment strategy for inducing deep remissions and improving outcomes in this specific subgroup.

**Methods:**

The BELI(E)VE trial (NCT05853965) is a German investigator-initiated, prospective, multicenter, open-label phase I/IIa study designed to evaluate the combination of belantamab mafodotin and venetoclax, with or without dexamethasone, in patients with relapsed or refractory multiple myeloma (RRMM) with ≥1 prior treatment line and t(11;14) translocation. The study consists of a dose escalation phase (phase I) to determine the recommended phase 2 dose (RP2D), followed by a dose expansion phase (phase IIa) to assess clinical activity at the RP2D. The phase I portion employs a traditional 3+3 design across four dose levels, while the phase IIa expansion will include 25 additional patients treated at the RP2D. Safety, tolerability, and preliminary efficacy will be evaluated through comprehensive assessments, including assessment for minimal residual disease negativity and ocular exams.

**Discussion:**

The BELI(E)VE trial aims to establish a novel personalized therapeutic approach for a specific subset of MM patients by combining belantamab mafodotin and venetoclax. This combination targets BCMA and BCL-2, potentially inducing deep and durable remissions. The study will provide critical data on the safety and efficacy of this combination, aiming to improve outcomes in t(11;14)-positive RRMM patients. The translational component, examining circulating tumor cells and mitochondrial activity, may identify mechanisms of resistance and response, guiding future personalized therapeutic strategies.

**Trial registration:**

NCT05853965 (2023-04-22).

**Supplementary Information:**

The online version contains supplementary material available at https://doi.org/10.1186/s12885-026-16905-3.

## Background

Multiple myeloma (MM) is a widely incurable B-cell malignancy that accounts for 10% of all hematological malignancies and is the most prevalent hematologic malignancy in patients older than 65 years [1].

Treatment paradigms and outcomes for patients with MM have changed substantially over the past decade with the introduction of effective and tolerable proteasome inhibitor (PI), immunomodulatory agent (IMiD), anti-CD38, and T-cell-redirecting therapies. Recent real-world data from European registries report pooled median overall survival (OS) of 85.5 months across 2012–2023; median OS not yet reached in the 2020–2023 frontline sub-cohort [2]. Nevertheless, a significant unmet need remains for the treatment of relapsed and/or refractory MM (RRMM). Patients who are triple-class exposed (having received at least one PI, one IMiD and a monoclonal anti-CD38 antibody) had an impaired prognosis with a median OS of 13.8 months; and median OS was worse for those refractory to all three classes with 8.6 months [3–5]. Penta-refractory patients (refractory to two PIs, two IMiDs, and an anti-CD38 antibody) have a documented median OS of less than six months [4]. These data underline the unmet need for developing novel treatment options, especially in this pre-exposed patient group.

The prognosis of patients in first-line and relapse treatment is remission-dependent. Achievement of deep remissions is associated with superior outcomes. More recently, emerging data show that achieving minimal residual disease (MRD) negative remissions directly and reliably translates into better progression-free survival (PFS) and OS [6–8]. Hence, MRD negativity has recently been recommended as an early endpoint in MM clinical trials by the Oncologic Drugs Advisory Committee (ODAC) of the U.S. Food and Drug Administration (FDA) [9].

Besides the induction of MRD-negative remissions, the way towards personalized treatment approaches remains an unmet need. Currently, there are no approved biomarker-based therapeutic approaches in the management of MM. Given the multiple available drugs and combination regimens in this disease, predictive biomarkers to guide the selection of therapy would be an essential advancement in the care of these patients, delivering the most effective treatments to the appropriate patient population.

The BELI(E)VE trial therefore moves beyond the prevailing one-size-fits-all sequencing paradigm in myeloma and prospectively tests a genotype-directed combination in a molecularly defined subgroup. To our knowledge, BELI(E)VE is among the first investigator-initiated trials to combine an antibody-drug conjugate with a BCL-2 inhibitor specifically in t(11;14)-positive disease.

### Targeting BCMA and BCL-2 in MM

BCMA (B Cell Maturation Antigen), also designated as tumor necrosis factor receptor superfamily member 17 (TNFRSF17), is expressed on the surface of normal and malignant B lymphocytes [10] and plays an essential role in the development and survival of long-lived bone marrow plasma cells [11]. The restricted expression profile of BCMA makes it an excellent target for a therapeutic antibody with direct cell-killing activity and has limited off-target effects [12]. BCMA-targeted therapies demonstrated impressive results with BCMA-targeted Chimeric Antigen Receptor T cell therapy (CAR-T) [13, 14], BCMA × CD3 bispecific antibodies [15–17], and antibody-drug conjugate (ADC) belantamab mafodotin [18–22]; a total of six BCMA-targeted drugs (2 CAR-T cell constructs, three bispecific antibodies, and one ADC) had received approval in the European Union and the United States for the treatment of relapsed and/or refractory MM (RRMM). While the conditional approval of the ADC belantamab mafodotin was withdrawn following the non-superiority in the DREAMM-3 trial, full approval was granted by EMA in July 2025 based on the significant superiority of belantamab mafodotin in combination with bortezomib and dexamethasone (BVd; median (m)PFS 36.6 months) over daratumumab plus bortezomib and dexamethasone (DVd; mPFS 13.4 months), including an OS benefit [21], and in combination with pomalidomide and dexamethasone (BPd, mPFS not reached) over pomalidomide, bortezomib, and dexamethasone (PVd, mPFS 12.7 months) [22].

**Fig. 1:**
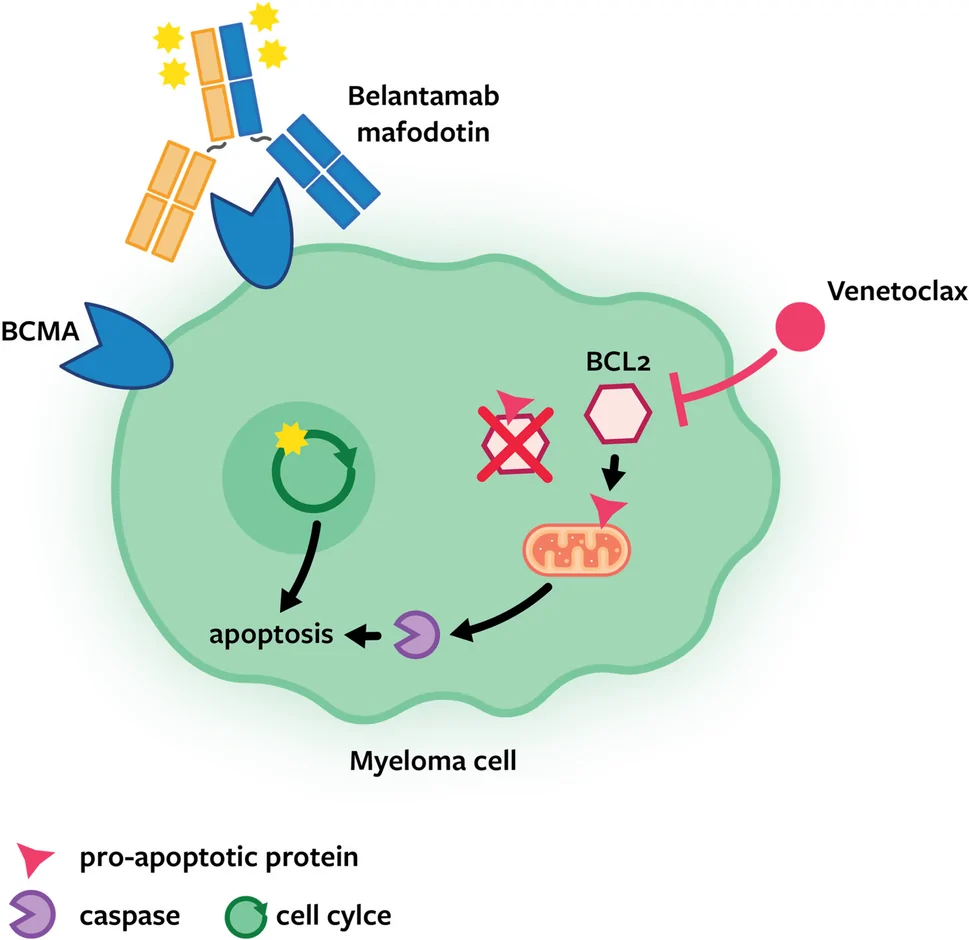
Modes of action of Belantamab mafodotin and Venetoclax: Belantamab mafodotin targets BCMA on the surface of myeloma cells, leading to its internalization. Subsequently, the cytotoxin mcMMAF is released within the cell, interacting with microtubules by binding to tubulin. This interaction results in cell cycle arrest between the G2 and M phases, ultimately inducing cell death through apoptosis. On the other hand, venetoclax selectively inhibits the Bcl-2 protein, which is overexpressed in t(11;14) positive myeloma cells. Venetoclax’s blockade of Bcl-2 facilitates the programmed cell death of these tumor cells

Overexpression of BCL-2 (B-cell lymphoma 2) significantly contributes to the pathogenesis of lymphoid malignancies, including MM [23]. Plasma cells are typically geared towards long-term survival and have low apoptotic rates associated with high levels of anti-apoptotic proteins such as BCL-2 and myeloid-cell leukemia (MCL)-1. Dysregulation of apoptotic pathways in these cells, often from aberrant BCL-2 and/or MCL-1 overexpression, is thought to play a significant role in the development and progression of MM. Mainly, myeloma cells with chromosomal translocation t(11;14) express high levels of BCL-2 relative to BCL-X_L_ and MCL-1 [23]. Antagonizing BCL-2 function to induce apoptosis has successfully been done in other hematologic malignancies and is also a compelling therapeutic approach in MM [23–25]. Venetoclax, a potent, orally bioavailable, small molecule inhibitor of the anti-apoptotic protein BCL-2, which restores programmed cell death (apoptosis) in cancer cells, showed durable responses in t(11;14)-positive MM patients in combination with bortezomib and dexamethasone (Vd) [26, 27]. The t(11;14) abnormality is seen in 15% to 20% of myeloma patients and, once present, is stably found over the course of the disease [28–30]. The t(11;14) is currently classified as a standard-risk genetic marker, although it is frequently found in plasma cell leukemia [28–30]. Additionally, dexamethasone, a steroid commonly used in MM treatment, upregulates the expression of the pro-apoptotic molecule BIM (BCL-2 -interacting mediator of cell death) and increases its binding to BCL-2, which results in increased sensitivity to venetoclax [23]. Therefore, combining a potent BCL-2 inhibitor (venetoclax) with low-dose dexamethasone in patients with t(11;14)-positive MM may lead to enhanced antitumor effects given the anticipated synergism between these two drugs (Fig. 1) [31].

Both belantamab mafodotin and venetoclax have been and are being investigated in clinical trials in patients with RRMM as monotherapy or in various treatment combinations. However, the combined treatment of an anti-BCMA-directed agent such as belantamab mafodotin with a BCL-2 inhibitor such as venetoclax has yet to be investigated. As both belantamab mafodotin and venetoclax, combined with dexamethasone to further enhance the anti-myeloma effect as depicted above, are candidate treatments inducing deep and long-lasting remissions, including MRD-negativity, the BELI(E)VE trial will evaluate the combination of belantamab mafodotin and venetoclax with and without dexamethasone in t(11;14)-positive RRMM. 

## Methods: study design, patient population, objectives, treatment, and control groups

The BELI(E)VE-Trial (NCT05853965) is an investigator-initiated, prospective, multicenter, open-label phase I/IIa study that consists of a dose escalation phase (phase I) and a dose expansion phase (phase IIa). Up to 49 patients (12–24 in Phase I, 25 in Phase II) are planned for total trial duration over 44 months. Survival follow-up lasts until last patient last visit of the study. Participating hospitals are in Germany and are listed in Supplementary Table S1.

### Patient population

The BELI(E)VE trial recruits adult patients with RRMM and the cytogenetic aberration of translocation t(11;14) due to their higher dependency on BCL-2. Initially, patients were required to have undergone at least four prior treatment lines (in line with the original belantamab mafodotin conditional approval), however following emerging data on the safety and efficacy of belantamab mafodotin combinations in early relapse [22, 32], the trial was amended, and now includes patients from first relapse on (one prior treatment line) who have been exposed to at least one proteasome inhibitor, one immunomodulatory agent and one anti CD38 monoclonal antibody. In addition, patients must have documented evidence of progressive disease on or after the last treatment line and sufficient organ function, as defined in Table 1.

**Table 1:** Eligibility (inclusion and exclusion) criteria

Inclusion criteria	Exclusion criteria
1. Subjects must be ≥ 18 years of age.	1. Subject has received prior Venetoclax and/or anti BCMA treatment.
2. Subjects must have an Eastern Cooperative Oncology Group (ECOG) performance status of ≤ 2	2. Participant has used an investigational drug or approved systemic anti-myeloma therapy within 14 days or five half-lives, whichever is shorter, preceding the first dose of study drug. The only exception is emergency use of a short course of corticosteroids (equivalent of Dexamethasone 40 mg/day for a maximum of 4 days) up to 7 days before treatment.
3. Subjects must voluntarily sign and date an informed consent form	3. Participant has had plasmapheresis or radiation therapy within 7 days prior to first dose of study treatment
4. Subjects must have had documented multiple myeloma requiring treatment as defined by the criteria below:Monoclonal plasma cells in the bone marrow > 10% and/or presence of a biopsy proven plasmacytoma at some point in their disease history requiring treatment according to diagnostic criteria (IMWG updated criteria 2014, Rajkumar et al. 2014) with measurable disease at screening (serum M-protein > 500 mg/dL or urine M protein 200 mg/24h, in case of oligosecretory MM serum free light chain > 10mg/dL and abnormal kappa/lambda free light chain ratio)	4. Participant has current corneal epithelial disease except mild changes in corneal epithelium
5. Cytogenetics/FISH confirming t(11;14)	5. Participant has current unstable liver or biliary disease defined by the presence of ascites, encephalopathy, coagulopathy, hypoalbuminemia, esophageal or gastric varices, persistent jaundice, or cirrhosis.Note:Stable non-cirrhotic chronic liver disease (including Gilbert’s syndrome or asymptomatic gallstones) or hepatobiliary involvement of malignancy is acceptable if otherwise meets entry criteria.
6. Prior treatment requirements: - Subjects must have received at least 1 prior treatment line (induction, high-dose, consolidation and maintenance is considered as one treatment line). All patients must have received at least one proteasome inhibitor and at least one immunomodulatory agent and at least one anti CD38 monoclonal antibody. - Subjects must have documented evidence of progressive disease on or after the last treatment line.- Subjects with a history of autologous SCT are eligible for study participation provided the following eligibility criteria are met: i. ASCT was >100 days prior to initiating study treatment, and ii. No active bacterial, viral, or fungal infection(s) present.	6. Participant has a presence of active renal condition (infection, requirement for dialysis or any other condition that could affect participant’s safety). Participants with isolated proteinuria resulting from MM are eligible, provided they fulfil inclusion criteria
7. Subjects must have adequate organ function, defined as follows: a. Hemoglobin ≥8.0 g/dL (without transfusion of red blood cells for the past 14 days) b. Absolute neutrophil count ≥ 1.5 x 10^9^/L (without growth factor support for the past 14 days) c. Platelet count more or equal 75 x 10^9^/L (without growth factor or platelet stimulating agents for the past 14 days) d. Adequate hepatic function per local laboratory reference range as follows: i. Aspartate aminotransferase (AST) ≤ 2.5 x upper limit of normal (ULN); ii. Alanine aminotransferase (ALT) ≤ 2.5 x ULN iii. Total bilirubin ≤ 1.5 x ULN, except in subjects with congenital bilirubinemia, such as Gilbert syndrome (direct bilirubin ≤ 1.5 x ULN).Isolated bilirubin ≥1.5xULN is acceptable if bilirubin is fractionated and direct bilirubin <35%. e. Subjects must have adequate renal function as demonstrated by eGFR ≥30 mL/min/ 1.73 m2 as calculated by Modified Diet in Renal Disease (MDRD) formula f. Spot urine (albumin/creatinine ratios (spot urine) <500 mg/g (56 mg/mmol) g. Corrected serum calcium ≤ 14 mg/dL (≤3.5 mmol/L); or free ionized calcium < 6.5 mg/dL (<1.6 mmol/L)	7. Participant has had major surgery ≤ 4 weeks prior to initiating study treatment. Kyphoplasty is not considered a major surgery.
8. A female participant is eligible to participate if she is not pregnant or breastfeeding, and at least one of the following conditions applies: a. Is not a woman of childbearing potential (WOCBP) b. Is a WOCBP and using a contraceptive method that is highly effective (with a failure rate of <1% per year), preferably with low user dependency (as described in Appendix 14.1.4) before and during the intervention period and for at least 5 months after the last dose of study intervention and agrees not to donate eggs (ova, oocytes) for the purpose of reproduction during this period. The investigator should evaluate the effectiveness of the contraceptive method in relationship to the first dose of study intervention. OR A WOCBP must have a negative highly sensitive serum pregnancy test (as required by local regulations) within 24 h before the first dose of study intervention and agree to use a highly effective method of contraception before and during the study and for 5 months after the last dose of IMP.	8. Participant must not use contact lenses while participating in this study. Bandage contacts may be prescribed by an eye care professional if needed.
9. Male participants are eligible to participate if they agree to the following during the intervention period and for 6 months after the last dose of study treatment to allow for clearance of any altered sperm: a. Refrain from donating sperm PLUS either: b. Be abstinent from heterosexual intercourse as their preferred and usual lifestyle (abstinent on a long term and persistent basis) and agree to remain abstinent. OR c. Must agree to use contraception/male condom plus partner's highly effective method, failure rate < 1% per year	9. Participant has any evidence of active mucosal or internal bleeding or other gastrointestinal disease that may significantly alter the absorption of oral drugs.
10. All subjects must agree to refrain from donating blood while on study drug and for 28 days after discontinuation from this study treatment.	10. Participant has evidence of cardiovascular risk including any of the following: a. Evidence of current clinically significant uncontrolled arrhythmias, including clinically significant ECG abnormalities such as 2nd degree (Mobitz Type II) or 3rd degree atrioventricular (AV) block b. History of myocardial infarction, acute coronary syndromes (including unstable angina), coronary angioplasty, or stenting or bypass grafting within three (3) months prior to Screening. c. Class III or IV heart failure as defined by the New York Heart Association functional classification system [NYHA, 1994] d. Uncontrolled hypertension e. Left ventricular Ejection Fraction ≤ 50%
11. All subjects must agree not to share study medication.	11. Participant has known immediate or delayed hypersensitivity reaction or idiosyncratic reactions to IMPs or drugs chemically related to IMPs, or any of the components of the study treatment
12. All prior treatment-related toxicities (defined by National Cancer Institute- Common Terminology Criteria for Adverse Events (NCI-CTCAE), version 5.0) must be ≤ Grade 1 at the time of enrolment except for alopecia.	12. Participant has an invasive malignancy other than disease under study within 5 years before trial inclusion, except o Adequately treated in situ carcinoma of the cervix uteri or the breast; o Basal cell carcinoma of the skin or localized squamous cell carcinoma of the skin; o Prostate cancer Gleason grade 6 or lower AND with stable Prostate Specific Antigen (PSA) levels off treatment; o Previous malignancy with no current evi-dence of disease, and which was confined and surgically resected (or treated with other modalities) with curative intent and unlikely to impact survival during the duration of the study.
	13. Participant is pregnant or lactating
	14. Participants who have had prior allogeneic stem cell transplant.
	15. Participants with symptomatic amyloidosis, active POEMS syndrome (polyneuropathy, organomegaly, endocrinopathy, monoclonal plasmaproliferative disorder, skin changes) or active plasma cell leukaemia at the time of screening.
	16. Participants with any serious and/or unstable pre-existing medical, psychiatric disorder or other conditions (including lab abnormalities) that could interfere with participant’s safety, obtaining informed consent or compliance to the study procedures.
	17. Subject is known to be seropositive for human immunodeficiency virus (HIV) or hepatitis B (defined by a positive test for hepatitis B surface antigen (HBsAg) or antibodies to hepatitis B surface and core antigen (anti HBs and anti HBc respectively), or hepatitis C (anti-HCV antibody positive or HCV RNA quantitation positive).
	18. Current immune or inflammatory conditions requiring immunosuppressive treatment (e.g. systemic lupus erythematosus, rheumatoid arthritis).
	19. Subject must not have received any live vaccines within 8 weeks prior to first dose of study treatment.
	20. Subject must not use or anticipate the use of prohibited medications or foods during study participation.
	21.Subject has a history of or show any signs of known meningeal/central nervous system involvement by myeloma.
	22. Evidence of other clinically significant uncontrolled condition(s) including, but not limited to: a. Uncontrolled and/or active systemic infection (viral, bacterial or fungal) requiring treatment. b. Any concurrent medical or psychiatric condition or disease (eg. uncontrolled diabetes, acute diffuse infiltrative pulmonary disease) that is likely to interfere with the study procedures or results, or that in the opinion of the investigator, would constitute a hazard for the participation in this study.
	23. Subject is known or suspected of not being able to comply with the study protocol (eg. because of alcoholism, drug dependency, or psychological disorder). Subject has any condition for which, in the opinion of the investigator, participation would not be in the best interest of the subject (e.g., compromise the well-being) or that could prevent, limit or confound the protocol-specified assessments.
	24. Treatment with any of the following within 7 days prior to the first dose of study drug: a. moderate or strong cytochrome P450 3A (CYP3A) inhibitors b. moderate or strong CYP3A inducers
	25. Administration or consumption of any of the following within 3 days prior to the first dose of study drug: a. grapefruit or grapefruit products b. Seville oranges (including marmalade containing Seville oranges) c. star fruit
	26. Participation in any other clinical trial (with the exclusion of observational studies)

The first patient was included in the BELI(E)VE trial in April 2024.

### Study objective

In the dose exploration phase I, the safety and tolerability profile of belantamab mafodotin in combination with venetoclax with or without dexamethasone will be evaluated in four different dose levels of the combination treatment. In case of sufficient tolerability and preliminary efficacy, phase I will then be followed by a phase IIa expansion to further evaluate the clinical activity of the combination treatment with the recommended phase 2 dose (RP2D).

The primary objective of the phase I trial part is the establishment of the maximum tolerated dose (MTD) and RP2D of treatment with venetoclax in combination with belantamab mafodotin with or without dexamethasone in patients with RRMM and t(11;14). Further, the safety profile of the combination treatment is comprehensively evaluated. In the subsequent phase IIa trial, the primary objective is assessing the safety and efficacy of the established recommended dose in an expansion cohort. The trial’s secondary objectives include assessing the overall response rate (ORR), MRD negativity rate, PFS, and duration of response (DOR) of the established recommended dose.

### Treatment and selection of doses in the study

**Fig. 2:**
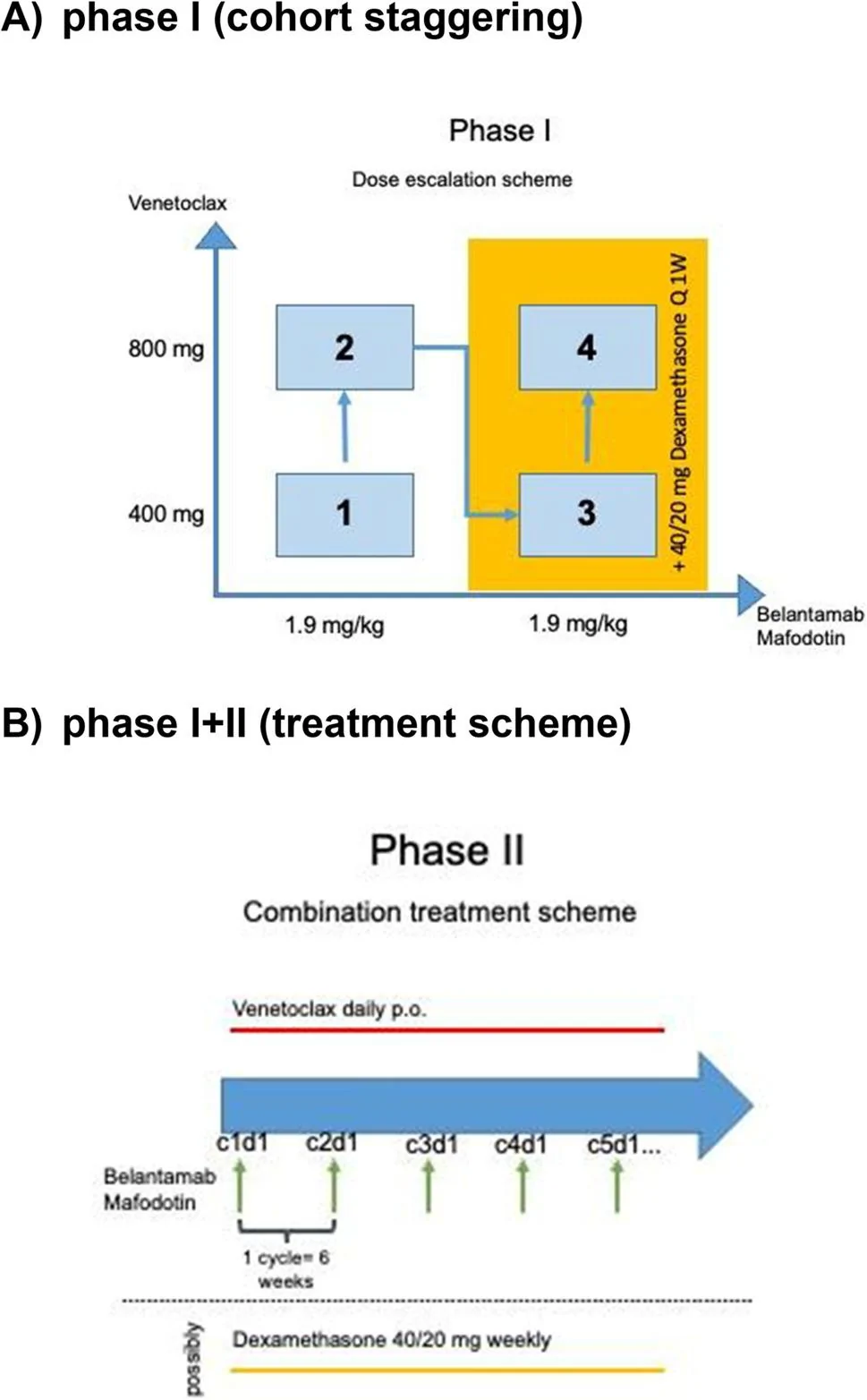
Trial timeline flow. **A** The dose escalation phase I follows the traditional 3 + 3 scheme. **B** The phase IIa combination treatment scheme includes Belantamab mafodotin, venetoclax, and possibly dexamethasone

The selected dosage regimen was chosen to sequentially assess the safety and efficacy of each dose level combination. The dose escalation phase I follows the traditional 3 + 3 scheme to establish the recommended treatment dose with venetoclax in combination with belantamab mafodotin with or without dexamethasone. The first combination cohort begins with the starting dose, and the remaining cohorts follow with the second to fourth dose, so ideally, 12 patients will be analyzed (Fig. 2). Dexamethasone is added in cohorts 3 and 4 to establish an optimized triplet combination.

The dosing regimen with belantamab mafodotin at 1.9 mg/kg IV every 6 weeks and venetoclax as an oral drug results in a low treatment burden and convenient regimen for patients (Fig. 1).

The reduced 1.9 mg/kg every 6 weeks schedule for belantamab mafodotin was based on emerging data showing a long half-life and durable responses of the substance and was selected to reduce the risk and severity of corneal toxicity while preserving anti-myeloma activity. This schedule represents a substantial de-escalation versus the Q3W schedule of DREAMM-7 and the Q4W schedule of DREAMM-8 [21, 22], which is supported by post-hoc analyses showing that extended dosing may reduce ocular adverse reactions [33]. Subsequent trials, including the phase 3 DREAMM-10 study (NCT06679101) of belantamab mafodotin–lenalidomide–dexamethasone in transplant-ineligible newly diagnosed myeloma, have prospectively adopted Q8W dosing for the first 24 weeks followed by Q12W thereafter [34]. The BELI(E)VE schedule was finalized before these data were available; further interval extension may be evaluated in subsequent studies.

Venetoclax will be given in escalating doses of 400 mg and 800 mg (PO, daily [QD]). Prior data has shown good safety profiles of up to 800 mg QD [26]; however, a lower dose of 400 mg will be assessed first in combination with belantamab mafodotin only.

In the next step, dexamethasone (40 mg PO; 20 mg for patients ≥ 75 years, weekly [Q1W]) will be added to the combination therapy, again starting with the lower dose of venetoclax. The fourth and last cohort of the phase I part will hence assess the combination of belantamab mafodotin (1.9 mg/kg IV Q6W), venetoclax (800 mg PO, QD), and dexamethasone (40/20 mg PO, Q1W) (Table 2).

Based on the results of the Phase I part, the data safety monitoring board (DSMB), consisting of an independent group of two leading experts on MM and one biostatistician, will recommend the dosage for the Phase IIa part (RP2D) (Table 2).

**Table 2:** Treatment schedule

Drug	Dose	Route	Days
**Starting Dose level, 6-week-cycle (Q6W)**			
Belantamab mafodotin	1.9 mg/kg	IV	D1
Venetoclax	400 mg	PO	daily (QD)
**Second Dose level, 6-week-cycle (Q6W)**			
Belantamab mafodotin	1.9 mg/kg	IV	D1
Venetoclax	800 mg	PO	daily (QD)
**Third Dose level, 6-week-cycle (Q6W)**			
Belantamab mafodotin	1.9 mg/kg	IV	D1
Venetoclax	400 mg	PO	daily (QD)
Dexamethasone	40 mg (20 mg for subjects ≥ 75 years)	PO	weekly (Q1W)
**Fourth Dose level, 6-week-cycle (Q6W)**			
Belantamab mafodotin	1.9 mg/kg	IV	D1
Venetoclax	800 mg	PO	daily (QD)
Dexamethasone	40 mg (20 mg for subjects ≥ 75 years)	PO	weekly (Q1W)

### Trial assessments

A baseline assessment is performed within 30 days before treatment (Table 3), including an ocular exam, skeletal survey, extramedullary plasmacytoma assessment, bone marrow biopsy, and measurable disease evaluation. During treatment, an assessment is performed as described in Table 3 for every cycle, including response to treatment evaluation. Bone marrow analysis will be done every fourth cycle (= every 6 months) and at progression, with a central laboratory assessing MRD status. At the end of the treatment (EOT) for each patient, a final assessment should be made according to Table 3. The follow-up will be continued as part of a non-interventional follow-up (Table 3). All patients will enter a follow-up period during which toxicity, remission state, and anti-myeloma treatment parameters will be documented.

**Table 3:** Study assessments

**Study Assessments**				
	Baseline	During treatment	EOT	Follow up ^17^
Informed Consent	X			
Inclusion/Exclusion criteria	X			
Demography	X			
Medical History (includes substance abuse)	X			
Full physical exam, Vital Signs (BP, HR, Body Temperature) and ECOG Performance Status	X	X	X	
Ocular Exam ^1^	X	X	X	X
Weight and Height	X	X	X	
Education about life style considerations for the study	X	X	X	
Past and current medical conditions	X			
Concomitant Medication	X	X	X	
Quality of Life Questionnaire	X	X	X	
Serum Pregnancy Test (WOCBP only) ^2^	X	X	X	X
Hematology ^3^	X	X	X	
Blood clinical chemistry, Thyroid function tests, Estimated Glomerular Filtration (eGFR) ^4,5,6^	X	X	X	
Urine Dipstick	X	X	X	
Spot Urine (albumin/creatinine ratio) or urine dipstick ^7^	X	X	X	
HIV, HBsAG, HBcAG, HCV test ^8^	X			
12-lead ECG ^9^	X	X	X	
Echocardiography (LVEF) ^10^	X	X#	X#	
ß2 Microglobulin	X	X	X	
UPEP 24 h urine collection	X	X	X	
Urine immunofixation	X	X	X	
SPEP ^11^	X	X	X	
serum immunofixation	X	X	X	
serum FLC assay ^12^	X	X	X	
IgG, IgA (IgD or IgE, if applicable ^13^)	X	X	X	
Calcium corrected for albumin (serum)	X	X	X	
Skeletal survey (Whole-body Low dose Osteo CT) ^14^	X	X#		
Extramedullary Plasmocytoma Assessment (by whole body CT or whole-body MRI or CT/PET) ^15^	X	X	X	
BM (biopsy or aspirate) for local disease assessment	X	X	X	
BM aspirate (central lab) for cytogenetics/ FISH testing, MRD testing and CTC assessment^16^	X	X^16^	X	
Peripheral blood (central lab)	X	X	X	
Adverse Events ^18^		X	X	X
Ocular Prophylaxis		X		
Dosing of study medication		X		

X = perform

# = perform if clinically indicated

1. Screening ocular examination will be performed by an ophthalmologist (or optometrist, if an ophthalmologist is not available) within 30 days prior to C1D1 and shortly before dosing for the first 6 cycles (window allowed is up to 5 days prior to each dosing visit. All efforts should be made to schedule as close to Belantamab Mafodotin dosing as possible). After the 6th cycle of Belantamab Mafodotin, if there are no significant ocular symptoms or vision changes, the frequency of ophthalmic exams may be decreased to every 3 months until the end of treatment. In cases of persistent or newly developed ocular symptoms, the participants will have further ophthalmological exams at least every 3 months until resolution to grade 1 or baseline or more frequently as clinically indicated by the eye care specialist

2. Perform only in women of child-bearing potential. Two serum pregnancy tests should be performed at screening. The first test should be performed at least 10 days prior to C1D1 and the second test within 24 h prior to administration of C1D1. Further serum pregnancy tests should be performed within 48 h prior to each dose. Final pregnancy test (serum or, in this case, urine) must be performed in WOCBP at least 70 days after the last dose of IMP, preferably at a visit. Follow up pregnancy assessment by telephone (for WOCBP only) should be performed 4 months after the last dose of Belantamab Mafodotin

3. hematology: hemoglobin, leukocyte count (differential count), platelets

4. blood chemistry: total proteins, albumin, creatinine, GOT/AST, GPT/ALT, γ-GT, urea, total bilirubin, alkaline phosphatase, LDH, CRP, calcium, sodium, potassium, uric acid, serum glucose

5. TSH, free T3 and T4

6. eGFR as calculated by Modified Diet in Renal Disease (MDRD) formula

7. Urine dipstick for protein may be used to assess for presence of urine protein. Albumin/creatinine ratio needs to be done in any participant with urine dipstick result of ≥ 1 + at screening, or with positive protein if urine dipstick protein quantification is not available

8. Hep C RNA testing is optional, but it may be performed to determine participant eligibility if Hep C antibody positive. If negative, patient is eligible

9. ECG on C1d1 + d8 and C2d1 only

10. ECHO may be performed within 30 days prior to C1D1

11. SPEP and UPEP will include M protein calculations

12. Serum Free Light Chain assay will include kappa/lambda ratio and quantification of involved and uninvolved light chains

13. IgD/IgE testing is only required for patients with IgD/IgE myeloma

14. Skeletal survey results within 30 days prior to C1D1 may be used for screening. Same modality used at Screening should be used throughout study. During treatment skeletal Survey at the time of suspected disease progression or as clinically indicated. Same modality used at Screening should be used throughout study

15. In participants with known or suspected extramedullary plasmacytoma, a whole-body scan (i.e., CT, MRI, or PET-CT) should be performed within 30 days prior to C1D1. The same method should be used throughout the study. Selected target lesion needs to be measured and followed over time. Further extramedullary plasmocytoma assessment as clinically indicated, or to confirm PD, imaging with PET/CT or Osteo-CT or WB-MRI may be performed by the same method throughout the study as was done at baseline

16 BM analysis mandatory on Cycle 4d1, C8d1, C12d1 and at PD. BM analysis may be done within 5 days before the mentioned time points. At these defined time points, also peripheral blood samples need to be collected. Additional BM analyses shall be performed as necessary to confirm response status (e.g. CR/sCR) according to IMWG

17. The survival for MM including data for subsequent MM therapies and remission state will be documented in medical charts. No visit necessary. Contacts will be made via phone calls, emails or other means of communications every 12 weeks until disease progression. After disease progression, only survival or date and cause of death will be documented every 6 months until end of the trial. Participant does not need to come in for visit unless they are being followed for corneal signs that are present at the end of study treatment

18. AEs/SAEs will be collected up to 70 days after the last dose of study treatment. All AEs/SAEs will be followed until the event is resolved, stabilized, otherwise explained, or the participant is lost to follow-up

As indicated in Table 3, quality of life questionnaires EQ-5D, QLQ-C30, and QLQ-MY20 are collected from all participants at baseline and additional time points.

### Ocular toxicity prevention and management

Given the known risk of corneal events with belantamab mafodotin, the protocol mandates baseline ophthalmologic examination and pre-dose ophthalmologic assessment for the first 6 cycles, with subsequent assessments every 3 months in the absence of significant findings. All participants receive prophylactic preservative-free artificial tears (4–8 times daily from Cycle 1 Day 1 through the end of belantamab mafodotin treatment) and are offered cooling eye masks during infusion. Contact lenses are prohibited during study treatment. Belantamab mafodotin dose modifications (continuation, dose hold until resolution, or dose reduction) follow the Keratopathy Visual Acuity (KVA) Scale, integrating ophthalmologic examination findings, best-corrected visual acuity, and patient-reported symptoms. Participants with persistent corneal findings at EOT are followed monthly for up to 12 months or until full resolution of findings.

### Infection prevention

Anti-infective prophylaxis is recommended for all participants for at least the first 12 weeks of treatment and for at least 30 days following disease progression, using amoxicillin/clavulanic acid, levofloxacin, or an institution-specified equivalent. Antiviral (HSV/VZV) and *Pneumocystis jirovecii* (PJP) prophylaxis are administered throughout study treatment at investigator discretion per institutional guidelines.

### Study outcomes and endpoints

The primary endpoint is defined as (I) percentage (number) of patients with dose-limiting toxicities (DLTs) with DLTs defined as: (a) prolonged neutropenia grade 4 for more than ten days, (b) prolonged thrombocytopenia grade 4 for more than seven days, (c) any grade 4 non-hematologic toxicity, (d) and any grade 3 reported non-hematological toxicity, which is assigned with a possible relationship to the applied study drugs as reviewed by the Data Safety Monitoring Board (DSMB); and (II) the percentage (number) of participants with adverse events (AEs), changes in clinical signs and laboratory parameters. AEs will be graded according to Common Terminology Criteria of Adverse Events (CTCAE) v5.0.

Secondary efficacy endpoints are overall response rate (ORR), rates of IMWG responses, minimal residual disease (MRD) negativity (sensitivity 10^− 5^) rate, PFS, duration of response (DOR), and health-related quality of life assessments as a patient-reported outcome.

Assessment of circulating tumor cells (CTC), investigations of mitochondrial activity, and single-cell analyses for immunophenotyping will be investigated as exploratory biomarkers.

### Statistical considerations and data handling

The trial is designed as a phase I/IIa study to analyze the safety of venetoclax and belantamab mafodotin as the primary endpoint.

### Sample size

In Phase I of the dose escalation, we employ the standard 3 + 3 design to limit the number of patients exposed to the belantamab mafodotin and venetoclax combination and ensure safety. This approach involves forming cohorts of three patients each. The initial cohort receives the starting dose, followed by subsequent cohorts receiving the second through fourth doses, aiming to evaluate 12 patients (Fig. 2). Dexamethasone is included for cohorts three and four to optimize the triplet combination.

If none of the three patients in a cohort experiences dose-limiting toxicities (DLT), another three patients will be treated at the next higher dose level. However, if one of the first three patients experiences DLT, three more patients will be treated at the same dose level. For the dose escalation phase I, 12–24 patients will be allocated to the trial. The RP2D will be identified based on the safety and preliminary efficacy in phase I.

Phase I will be followed by a cohort expansion phase IIa, which will evaluate the clinical activity of the combination treatment with RP2D in 25 more patients.

### Statistical analyses

The full analysis set population and safety evaluation consists of all patients with at least one dose of venetoclax in combination with belantamab mafodotin (± dexamethasone) or any investigational medical product intake, respectively.

The primary and secondary endpoint analyses will be performed in the safety population, including a planned interim analysis regarding phase I patients. All analyses will be conducted descriptively and interpreted in an explorative way. Details for the statistical evaluation of the results will be performed according to the predefined statistical analysis plan (SAP).

### Trial conduct and dissemination

Data are collected using electronic case report forms, with source data monitored by the trial monitoring plan. This study strictly adheres to the principles outlined in Good Clinical Practice and complies with the European Union’s Clinical Trials Regulation No 536/2014. All trial-related information will be confidential and securely stored in paper and electronic formats. The independent DSMB conducts regular reviews of safety data to detect any potential safety issues or emerging trends.

The trial is registered at ClinicalTrials.gov (NCT05853965) and the EU Clinical Trials Registry (EudraCT 2021-001413-37; EU CT 2024-511794-30) and has obtained ethics approval from the leading ethics committee in Hamburg (Ref: 2023-100993-AMG-ff). Written informed consent from all participating patients is obtained before any study procedure at their respective recruitment hospital sites. Any necessary protocol amendments will be submitted to the competent authority and ethics committees and implemented at the sites upon approval.

Manuscripts detailing the results of the BELI(E)VE study will be submitted for publication in a peer-reviewed journal based on the phase I and phase IIa results and exploratory objectives. Recognition for the primary results will be attributed to all collaborators involved in the trial, in the form of authorship and contributorship, following the uniform requirements for authorship for manuscripts submitted to medical journals.

## Discussion

As triple-class refractory MM patients have a median progression-free survival of only a few months and a median overall survival of 8.6 months [3, 4], this group has an unmet need for new treatment options. Targeted therapies such as CAR T cell therapies and bispecific antibodies achieved deep and durable responses in this difficult-to-treat population; however, their availability is restricted to specialized centers, they come with a substantial economic burden, and not all MM patients qualify for these immunotherapies due to comorbidities. Belantamab mafodotin is a safe, easy-to-use BCMA-directed ADC with relatively low rates of off-tumor effects and can induce long-lasting responses. Venetoclax, an inhibitor of the anti-apoptotic protein BCL-2, which restores apoptosis in tumor cells, is an acknowledged option for MM patients with t(11;14) showing high response rates. This study evaluates a combination of two targeted agents, belantamab mafodotin and venetoclax, with and without dexamethasone in t(11;14)-positive RRMM.

Several phase 1/2 and 3 studies have been conducted in the MM indication with venetoclax. In the phase 1 study M13-367, 66 subjects with RRMM received daily venetoclax monotherapy at 300, 600, 900, or 1200 mg. In this study, subjects had a median of 5 prior therapies. The ORR was 21% (14/66), and 15% achieved a very good partial response or better (≥ VGPR) [27]. In patients with t(11;14)-positive MM, which comprised 46% (*N* = 30) of the study population, the ORR was 40%, with 27% of subjects achieving ≥ VGPR. A cohort of 20 patients with t(11;14) RRMM was further enrolled in the study and treated with venetoclax 800 mg/day plus oral dexamethasone 40 mg weekly (20 mg for patients ≥ 75 years of age). The overall response rate in this cohort was 60% (12/20) and included 7 VGPR and 6 PR [31].

The BELLINI study, a global phase 3 trial, evaluated the combination of bortezomib and dexamethasone with either venetoclax or placebo in patients with RRMM who were sensitive or naïve to proteasome inhibitors [26]. The study achieved its primary endpoint, showing a mPFS of 22.4 months for the venetoclax group versus 11.5 months for the control, with a hazard ratio (HR) of 0.63 (95% confidence interval [CI]: 0.44–0.90). It also reported significant improvements in ORR and VGPR rates in the venetoclax arm. Despite these promising results, the trial was stopped early due to an increased risk of death linked to venetoclax, including eight fatal infections, resulting in a lower OS rate (HR 2.03, 95% CI 1.04–3.95). However, subgroup analyses showed notable benefits for specific patients. Those with the t(11;14) genetic translocation had significant PFS improvement without a marked decrease in OS (PFS HR 0.11, 95% CI 0.02–0.56; OS HR 0.34, 95% CI 0.03–3.84). Patients with high BCL-2 gene expression also experienced better PFS (HR 0.50, 95% CI 0.29–0.86), though the effect on OS was less definitive (HR 1.45; 95% CI 0.57–3.68) [26]. These findings suggest that Venetoclax-based combination therapies may benefit patients with specific genetic profiles.

A dose-escalation phase 2 study evaluated the combination of venetoclax, carfilzomib, and dexamethasone in 49 patients with RRMM [35]. The ORR was 80%, and the CR rate was 41%. Among 13 patients with the t(11;14) translocation, 11 achieved at least a VGPR, with 7 attaining a CR. In this study, no DLTs were observed; the most common toxicities included diarrhea (65%), fatigue (47%), and nausea (47%). Grade 3 or 4 toxicities comprised hypertension (16%) and pneumonia (12%). Another recently published study, the M13-494 CANOVA trial, is the first study of combination therapy with venetoclax and dexamethasone compared with pomalidomide and dexamethasone exclusively in patients with t(11;14)-positive RRMM who have received ≥ 2 prior lines of therapy. While the CANOVA trial did not show a significant improvement in PFS for venetoclax compared to pomalidomide plus dexamethasone (9.9 vs. 5.8 months, *p* = 0.24), secondary endpoints such as ORR (62% vs. 35%) and rate of ≥VGPR (39% vs. 14%) were favorable [36]. Moreover, an ongoing phase I/II trial is investigating venetoclax in combination with daratumumab and dexamethasone, and preliminary results show deep, durable responses in patients with t(11;14) RRMM [37]. Belantamab mafodotin used as monotherapy was not associated with statistically improved PFS compared with pomalidomide and dexamethasone (PFS 11.2 versus 7 months; HR 1.03, 95% CI 0.72–1.47) in the DREAMM-3 trial [38]. However, in the recently reported data from the DREAMM-7 phase III study, belantamab mafodotin plus bortezomib and dexamethasone (Vd) showed a statistically significant PFS benefit versus daratumumab plus Vd (36.6 vs. 13.4 months), including an OS benefit [21]. Likewise, the belantamab mafodotin, pomalidomide, and dexamethasone triplet investigated in DREAMM-8 proved superior to pomalidomide, bortezomib, and dexamethasone [22]. These data underline the potency of belantamab mafodotin combinations in early relapse [21]. The realized dosing frequency of belantamab mafodotin in DREAMM-7 and DREAMM-8 was approximately every 8–12 weeks during prolonged treatment, supporting the longer-interval schedule selected for BELI(E)VE [33]. Furthermore, ongoing trials investigate the combination of belantamab mafodotin with partners such as bortezomib/lenalidomide/dexamethasone [39].

Regarding potential adverse events, no new safety signals using the combination of venetoclax and belantamab mafodotin are expected due to the different toxicity profiles of both well-known substances. In patients with MM, the most concerning adverse events of venetoclax include neutropenia and thrombocytopenia [27]. Moreover, gastrointestinal symptoms are common [27]. In contrast, belantamab mafodotin frequently causes corneal epithelium changes, manifesting as blurred vision, dry eyes, or other visual impairments in most patients. Thrombocytopenia is rarely reported during treatment with belantamab mafodotin [20]. Other common non-hematologic adverse reactions include nausea, pyrexia, infusion-related reactions, and fatigue [20]. To reduce the risk of keratopathy or corneal toxicity, the BELIEVE trial implemented a dosing regimen of 1.9 mg/kg intravenously every six weeks (Q6W), deviating from the initial approval of 2.5 mg/kg every three weeks (Q3W). This modification, informed by more recent data on the drug’s extended half-life and sustained efficacy, aims to improve patient tolerability [21, 22].

Combining two different targeted modes of action, belantamab mafodotin enabling anti-tumor activity of cells by antibody-dependent cellular cytotoxicity (ADCC) and ADC activity and venetoclax antagonizing BCL-2 function to induce apoptosis may induce deep and durable responses, including sustained MRD negativity, compared to single-agent regimens, as prolonged DOR was observed in patients who achieved a response to either drug in the respective studies [21, 22, 36]. With the broad and early use of the main drug classes of PIs, IMiDs, and anti-CD38-moAbs, treatment options with different modes of action, such as in the BELI(E)VE trial, are urgently needed to allow for a switch of drug classes. Furthermore, combining oral medication with less frequent intravenous administration in an outpatient setting enhances management efficiency, reduces costs, conserves healthcare resources, and improves patients’ quality of life.

In summary, the BELI(E)VE trial, combining belantamab mafodotin and venetoclax as two targeted therapies (± dexamethasone), may establish a new regimen candidate with potentially high rates of deep and durable responses and acceptable tolerability for RRMM patients with t(11;14).

## Supplementary Information

Supplementary Material 1.

Supplementary Material 2.

## Data Availability

No datasets were generated or analysed during the current study.

## Abbreviations

ADC: Antibody-drug conjugate
ADCC: Antibody-dependent cellular cytotoxicity
AE: Adverse event
ASCT: Autologous stem cell transplantation
BCL-2: B-cell lymphoma 2
BCL-XL: B-cell lymphoma extra-large
BCMA: B-cell maturation antigen
BPd: Belantamab mafodotin, pomalidomide and dexamethasone
BVd: Belantamab mafodotin, bortezomib and dexamethasone
C1: Cycle 1
CAR-T: Chimeric Antigen T cell therapy
CR: Complete remission
CTC: Circulating tumor cells
CTCAE: Common Terminology Criteria of Adverse Events
D1: Day 1
DLT: Dose-limiting toxicity
DOR: Duration of response
DSMB: Data safety monitoring board
DVd: Daratumumab, bortezomib and dexamethasone
ECOG: Eastern Cooperative Oncology Group (ECOG) Performance Status
eGFR: Estimated glomerular filtration rate
EOT: End of treatment
EU: European Union
FDA: U.S. Food and Drug Administration
FLC: Free Light Chain
IMiD: Immunomodulating agent
IMWG: International Myeloma Working Group
IV: Intravenous
kg: Kilogram
MCL-1: Myeloid-cell leukemia 1
mg: Milligram
MM: Multiple myeloma
MRD: Minimal residual disease
MTD: Maximum tolerated dose
ODAC: Oncologic Drugs Advisory Committee
ORR: Overall response rate
OS: Overall survival
Pd: Pomalidomide and dexamethasone
PFS: Progression-free survival
PI: Proteasome inhibitor
PJP: Pneumocystis jirovecii pneumonia
PO: Per os
PVd: Pomalidomide, bortezomib and dexamethasone
Q1W: Weekly
Q3W: Every three weeks
Q6W: Every six weeks
QD: Daily
RP2D: Recommended phase 2 dose
RRMM: Relapsed or refractory multiple myeloma
TNFRSF17: Tumor necrosis factor receptor superfamily member 17
Vd: Bortezomib and dexamethasone
VGPR: Very good partial response
VZV: Varicella-zoster virus
WOCBP: Woman of childbearing potential
