# Supplementary material for: Belantamab mafodotin plus venetoclax in t(11;14)-positive relapsed or refractory multiple myeloma – the BELI(E)VE trial
Source: BMC Cancer. 2026 Sep 16;26:1141. doi: 10.1186/s12885-026-16905-3 (PMC13602635; doi:10.1186/s12885-026-16905-3)
Supplement: Supplementary file 2 [file 12885_2026_16905_MOESM2_ESM.docx]

Table S1: Participating Trial Sites

Center
Location
Department of Hematology, Oncology and Bone Marrow Transplantation with Section Pneumology, University Medical Center Hamburg-Eppendorf
Hamburg, Germany
Department of Hematology and Oncology, University Hospital of Schleswig-Holstein Campus Lübeck and University of Lübeck
Lübeck, Germany
Department of Hematology, Oncology and Bone Marrow Transplantation, Klinikum Chemnitz
Chemnitz, Germany
Department III of Internal Medicine University Hospital of Ulm
Ulm, Germany
CCC-MV and Department of Internal Medicine C, University Medicine Greifswald
Greifswald, Germany
Department of Internal Medicine, Charité—University Medicine Berlin
Berlin, Germany
Internal Medicine V and National Center for Tumor Diseases, University Hospital Heidelberg
Heidelberg, Germany
Department I of Internal Medicine, University Hospital of Cologne
Cologne, Germany
Department of Hematology, Cellular Therapy, Hemostaseology and Infectiology University Hospital Leipzig
Leipzig, Germany
